# *In Silico* Assessment of Efficacy and Safety of I_Kur_ Inhibitors in Chronic Atrial Fibrillation: Role of Kinetics and State-Dependence of Drug Binding

**DOI:** 10.3389/fphar.2017.00799

**Published:** 2017-11-07

**Authors:** Nicholas Ellinwood, Dobromir Dobrev, Stefano Morotti, Eleonora Grandi

**Affiliations:** ^1^Department of Pharmacology, University of California, Davis, Davis, CA, United States; ^2^West German Heart and Vascular Center, Institute of Pharmacology, University Duisburg-Essen, Essen, Germany

**Keywords:** ultra-rapid delayed-rectifier K^+^ current, atrial fibrillation, mathematical modeling, ion channel blockers

## Abstract

Current pharmacological therapy against atrial fibrillation (AF), the most common cardiac arrhythmia, is limited by moderate efficacy and adverse side effects including ventricular proarrhythmia and organ toxicity. One way to circumvent the former is to target ion channels that are predominantly expressed in atria vs. ventricles, such as K_V_1.5, carrying the ultra-rapid delayed-rectifier K^+^ current (I_Kur_). Recently, we used an *in silico* strategy to define optimal K_V_1.5-targeting drug characteristics, including kinetics and state-dependent binding, that maximize AF-selectivity in human atrial cardiomyocytes in normal sinus rhythm (nSR). However, because of evidence for I_Kur_ being strongly diminished in long-standing persistent (chronic) AF (cAF), the therapeutic potential of drugs targeting I_Kur_ may be limited in cAF patients. Here, we sought to simulate the efficacy (and safety) of I_Kur_ inhibitors in cAF conditions. To this end, we utilized sensitivity analysis of our human atrial cardiomyocyte model to assess the importance of I_Kur_ for atrial cardiomyocyte electrophysiological properties, simulated hundreds of theoretical drugs to reveal those exhibiting anti-AF selectivity, and compared the results obtained in cAF with those in nSR. We found that despite being downregulated, I_Kur_ contributes more prominently to action potential (AP) and effective refractory period (ERP) duration in cAF vs. nSR, with ideal drugs improving atrial electrophysiology (e.g., ERP prolongation) more in cAF than in nSR. Notably, the trajectory of the AP during cAF is such that more I_Kur_ is available during the more depolarized plateau potential. Furthermore, I_Kur_ block in cAF has less cardiotoxic effects (e.g., AP duration not exceeding nSR values) and can increase Ca^2+^ transient amplitude thereby enhancing atrial contractility. We propose that *in silico* strategies such as that presented here should be combined with *in vitro* and *in vivo* assays to validate model predictions and facilitate the ongoing search for novel agents against AF.

## Introduction

Atrial fibrillation (AF) is characterized by rapid, irregular heart contractions following fast, disorganized electrical signals in the atria. AF is the most common cardiac arrhythmia, occurring in 1–2% of the general population and projected to increase dramatically in the coming decades (to 4% by 2050) with an aging westernized population (Andrade et al., 2014). The most effective current treatment for preventing recurrence of AF in the clinic is radiofrequency ablation. Pharmacological therapy against AF is limited by low efficacy and substantial adverse side effects including an increased risk of lethal ventricular tachyarrhythmias.

To maximize efficacy and minimize proarrhythmic risk, an AF-selective drug should exert potent effects on fibrillating atria without significantly impacting ventricular tissue function during normal sinus rhythm (nSR) (Ehrlich et al., 2008; Van Wagoner et al., 2015). A potential strategy to achieve this goal is to target ion channels that are predominantly expressed in atria vs. ventricles, such as K_V_1.5, carrying the ultra-rapid delayed-rectifier K^+^ current (I_Kur_). Genetic mutations causing both loss- and gain-of-function of I_Kur_ have been associated with atrial arrhythmias in human (Olson et al., 2006; Christophersen et al., 2013; Colman et al., 2017). In a previous investigation, we used an *in silico* strategy to define optimal K_V_1.5-targeting drug characteristics, including kinetics and state-dependent binding, that maximize AF-selectivity (i.e., fast pacing-rate selectivity) in human atrial cardiomyocytes (Ellinwood et al., 2017). Because this work was conducted in atrial cardiomyocytes under nSR conditions, the best-performing drug properties identified would have relevance for patients with paroxysmal AF that have not undergone extensive AF-related electrical remodeling (Grandi et al., 2012; Nattel and Dobrev, 2016).

Building on our previously established simulation framework, the major goal of this investigation was to determine the optimal drug characteristics of I_Kur_ inhibitors in long-standing persistent (chronic) AF (cAF) conditions. Although not a universal finding (Yue et al., 1997; Bosch et al., 1999; Grammer et al., 2000; Workman et al., 2001), previous reports showed that I_Kur_ is strongly diminished in cAF patients (Van Wagoner et al., 1997; Brandt et al., 2000; Van Wagoner and Nerbonne, 2000; Dobrev and Ravens, 2003; Christ et al., 2008; Caballero et al., 2010), making the therapeutic potential of inhibitors targeting this current uncertain (Ravens et al., 2013; Grandi and Maleckar, 2016). Indeed, evidence of anti-arrhythmic efficacy of K_V_1.5 inhibitors in clinical trials is lacking (Ravens et al., 2013). However, recent studies have suggested an anti-arrhythmic potential of I_Kur_-targeting drugs in cAF (Christ et al., 2008; Ford et al., 2013, 2016; Loose et al., 2014), as they can prolong action potential (AP) and effective refractory period (ERP) in atrial cardiomyocytes of cAF patients. Moreover, experimental evidence suggests that block of I_Kur_ enhances force of contraction of isolated human atrial trabeculae in cAF (Wettwer et al., 2004; Schotten et al., 2007). Our human atrial cardiomyocyte model confirmed that block of I_Kur_ results in prolongation and elevation of the AP plateau, which augments the Ca^2+^ transient (CaT) amplitude (CaT_amp_), thereby eliciting a positive inotropic effect (Grandi et al., 2011). Thus, I_Kur_ might be a useful atrial-selective target to potentially prevent reentry and related atrial hypocontractility in cAF. We propose that our computational approach, combined with *in vivo* and *in vitro* validation, might be useful to facilitate the identification of atrial-selective anti-arrhythmic drugs against AF (Bers and Grandi, 2011; Grandi and Maleckar, 2016).

## Methods

### Atrial AP model and simulations

**Figure 1:**
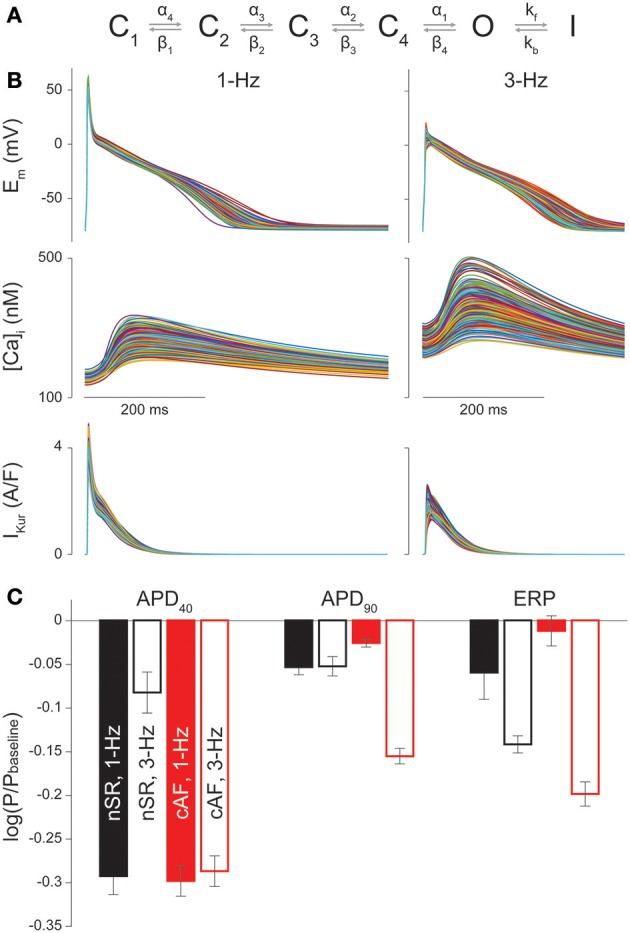
Sensitivity of nSR and cAF cardiomyocyte electrophysiology to I_Kur_ changes. **(A)** Drug-free Markov model of I_Kur_ derived from Zhou et al. (2012). The model has 4 closed states (C_1_, C_2_, C_3_, C_4_), a conducting open state (O), and an inactivated state (I). Transition rates equations and parameters are those in Ellinwood et al. (2017). **(B)** AP, CaT, and I_Kur_ in a subset of 300 cAF cardiomyocyte model variants at 1-Hz and 3-Hz pacing rates. **(C)** Bar graphs show the average regression coefficients indicating how perturbations in G_Kur_ affect APD_40_, APD_90_, and ERP in nSR and cAF at 1-Hz and 3-Hz pacing rates. Error bars represent one standard deviation.

APs and CaTs were simulated with the Grandi et al. model of the human atrial cardiomyocyte in nSR and cAF (Grandi et al., 2011; Morotti et al., 2016b). I_Kur_ gating was described by a 6-state Markov type model (Figure 1A) as in Ellinwood et al. (2017), and I_Kur_ maximal conductance (G_Kur_) in cAF was reduced by 50% compared to nSR (Grandi et al., 2011).

Simulations were equilibrated for 300 beats at 1-Hz pacing or 900 beats at 3-Hz pacing. After the 300^th^ or 900^th^ beat, the time to 40 and 90% repolarization of the AP (APD_40_ and APD_90_) were calculated, along with diastolic intracellular Ca^2+^ concentration ([Ca^2+^]_i_), CaT_amp_ and time to 50% CaT decay. The atrial ERP was determined using a standard S_1_-S_2_ premature stimulation protocol (Wang et al., 1996; Shinagawa et al., 2000; Christ et al., 2008; Zhao et al., 2009), where the S_1_ basal stimulus (5 ms in duration) was applied to a steady-state human atrial cardiomyocyte model. As previously described (Ellinwood et al., 2017), ERP was determined by applying the premature S_2_ stimulus (5 ms in duration, 2-fold the diastolic threshold of excitation) at progressively smaller S_1_-S_2_ intervals from 700 ms to refractoriness by decrements of 2 ms. The longest S_1_-S_2_ interval that failed to elicit an AP was taken as the local ERP (i.e., maximum upstroke velocity ≥5 V/s and AP with an amplitude ≥50% of the amplitude of the preceding AP elicited by S_1_).

An irregular pacing protocol was run for 20 s, starting from steady-state conditions at the fixed 3-Hz pacing. The cycle length (CL) was allowed to vary randomly following a uniform distribution between 285.7 and 400 ms, corresponding to a minimum pacing frequency of 2.5 Hz and a maximum pacing frequency of 3.5 Hz, with a mean of 333.3 ms (corresponding to 3-Hz pacing). The time course of membrane potential (E_m_), APD_90_, and CL was tracked over the course of the simulation.

All simulations and analysis were performed in MATLAB (The MathWorks, Natick, MA, USA) using the stiff ordinary differential equation solver ode15s. The model code is available for download at the following webpages: https://somapp.ucdmc.ucdavis.edu/Pharmacology/bers/ and http://elegrandi.wixsite.com/grandilab/downloads.

### Parameter sensitivity analysis

Parameter sensitivity analysis was performed with the population-based approach described in Sobie (2009), Morotti et al. (2017), and Morotti and Grandi (2017) to investigate the role of various currents and transporters in the regulation of AP duration (APD), ERP, and CaT characteristics. Two populations of 900 atrial cardiomyocyte models were generated by randomly varying the values of 18 parameters (see list in the Supplementary Materials) in the baseline nSR and cAF models. Specifically, the default value of each conductance or maximal transport rate was independently varied with a log-normal distribution (with standard deviation of 0.1). Multivariable regression (non-linear iterative partial least squares method) on log-transformed values was performed for 30 random subsets of 300 model variants from the 900-variant population to correlate the variation in each parameter to the consequent effect on each output. In Figure 1C and Figures S1–S6 bars represent the mean regression coefficients and error bars represent one standard deviation.

### K_V_1.5 drug-binding model

**Figure 2:**
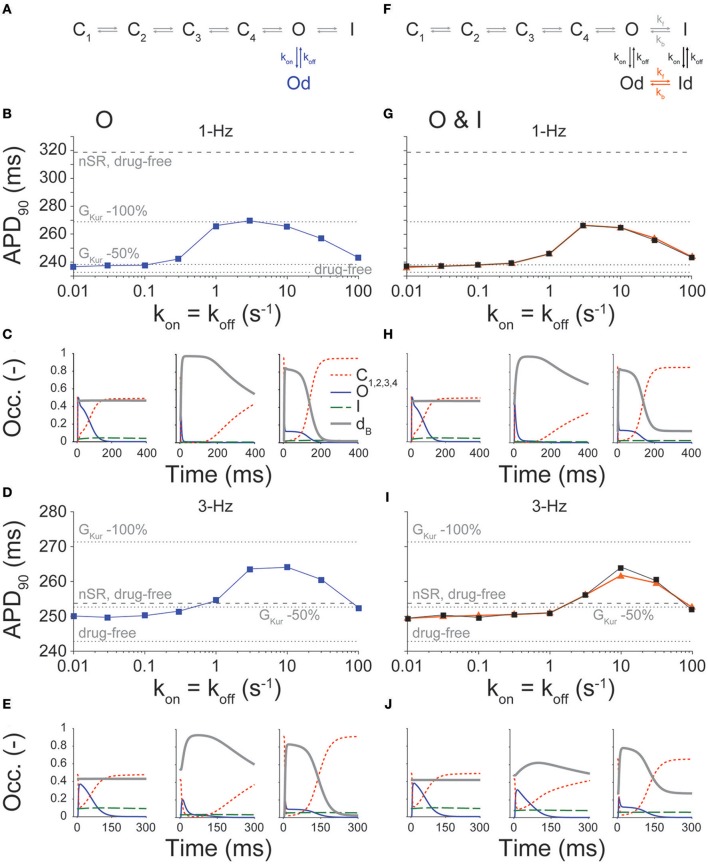
Effect of state-dependence and kinetics of drug binding on APD_90_. APD_90_ was determined for open (schematic in **A**, **B**, 1-Hz and **D**, 3-Hz pacing rate) and open and inactivated (schematic in **F**, **G** 1-Hz and **I**, 3-Hz pacing rate) state blockers given nine different rates of binding kinetics between 0.01 and 100 s^−1^ using half-logarithmic increments, whereby k_off_ = k_on_, K_d_ = 1 μM. For O & I blockers, we either allowed or prevented transitions between drug-bound states (orange vs. black traces in **G,I**). Simulations were also run in nSR and cAF drug-free conditions, and in cAF given a 50 and 100% reduction in G_Kur_ (dotted and dashed lines in **B**,**D**,**G**,**I**). Simulations were equilibrated for 300 beats at 1-Hz pacing or 900 beats at 3-Hz pacing using a [drug] equal to the IC_50_ value. **(C**,**E**,**H**,**J)** show the closed, open, inactivated and drug-bound (d_B_, i.e., Od or Od+Id) state occupancies during an AP for three different drug-binding kinetics (k_off_ = k_on_ = 0.01, 3, and 100 s^−1^).

We utilized our recent I_Kur_ Markov formulation and approach to describe various drug-K_V_1.5 channel binding schemes (Figures 2A,F; Ellinwood et al., 2017), as done by Lee et al. (2016). We previously showed that open state (O) blockers and open and inactivated state (O & I) blockers that target K_V_1.5 display fast pacing-rate selectivity (Ellinwood et al., 2017). Thus, we focused on these two types of inhibitors when examining the relationship between electrophysiological parameters and drug-binding kinetics in cAF. We considered different theoretical drugs with variable forward (k_on_) and reverse (k_off_) drug-binding rates to the open and inactivated states of the K_V_1.5 channel in the predicted physiological range of 0.01–100 s^−1^ (Lagrutta et al., 2006) using half-logarithmic increments resulting in nine transition rates for each drug state transition (0.01, 0.03, 0.1, 0.3, 1, 3, 10, 30, 100 s^−1^). For a particular state of the channel, dissociation constants (K_d_) for our drug scenarios were calculated as k_off_/k_on_, and affinity constants were calculated as k_on_/k_off_. To investigate the effects of these drug characteristics, for a given state-dependent binding inhibitor, we varied k_on_ and k_off_ together (k_on_ = k_off_) or considered all permutations of the nine different rates of drug binding (producing a total of 81 different drug scenarios). For drugs that could bind to multiple states of the K_V_1.5 channel, we also varied the relative affinity to open (K_O_) vs. inactivated state (K_I_). For O & I blockers, we included transitions between drug-bound states (orange transitions in Figure 2F) when specified. All drugs were simulated at the concentration causing a 50% reduction in peak I_Kur_ (i.e., IC_50_). IC_50_ values were computed as described previously (Ellinwood et al., 2017), using a 200-ms down-ramp voltage-clamp protocol from +30 to −60 mV. After the application of a given [drug] (range: 1 nM−1 M), we allowed sufficient time for the degree of block to reach equilibrium. IC_50_ values were calculated at 1- and 3-Hz pacing rates as the [drug] causing a 50% reduction in peak I_Kur_ compared to drug-free conditions. We chose the down-ramp, as compared to a typical square pulse, because it more closely resembles the relative state occupancies of the closed states, open state, and inactivated state of the K_V_1.5 channel during a physiological atrial AP, as we have shown in (Ellinwood et al., 2017).

## Results

### Role of I_Kur_ in nSR and cAF atrial electrophysiology

We built 900 variations of our nSR and cAF human atrial cardiomyocyte models (Grandi et al., 2011) at 1- and 3-Hz pacing, and performed parameter sensitivity analysis on 30 random subsets of 300 model variants to determine how alterations in each maximum ionic conductance/transport rate differentially (in cAF vs. nSR) affect electrophysiological properties including APD_40_, APD_90_, ERP, CaT_amp_, diastolic [Ca^2+^]_i_, and time to 50% CaT decay (Figures S1–S6). Simulated APs and CaTs in a representative group of 300 cAF cardiomyocyte model variants are shown in Figure 1B, and the average regression coefficients for G_Kur_ in nSR and cAF conditions at 1- and 3-Hz pacing are in Figure 1C. The values are negative because an increase in I_Kur_ shortens APD_40_, APD_90_, and ERP on average according to the regression algorithm. The analysis revealed that, while at a slow pacing rate APD_90_ and ERP are more sensitive to changes in G_Kur_, APD_40_ is similarly sensitive in nSR and cAF (Figure 1C). At 3-Hz pacing, G_Kur_ impacts AP and ERP prolongation more in cAF vs. nSR despite the fact that G_Kur_ is smaller in cAF conditions. This points to I_Kur_ inhibition as a promising approach to counteract the abbreviated APD and ERP in cAF, while having a more moderate effect at physiological pacing rates. Therefore, we next ran simulations to reveal I_Kur_-targeting drug properties that exhibit anti-AF selectivity and efficacy along with minimized proarrhythmic risk in cAF.

### Effect of conformational state specificity and binding/unbinding kinetics on human atrial cardiomyocyte APD at normal and fast pacing rates in cAF conditions

Figure 2 shows changes in APD caused by O and O & I inhibitors at varying drug-binding kinetics, whereby k_on_ is set equal to k_off_ (i.e., K_d_ = 1 μM). These are compared to no block, 50, and 100% reduction in G_Kur_ in cAF conditions, as well as no block in nSR conditions. Similar to our findings in nSR (Ellinwood et al., 2017), both types of inhibitors display a biphasic relationship between APD and drug-binding kinetics at 1- and 3-Hz pacing. At 1-Hz pacing, APD in the presence of drug is comparable to a 50% reduction in G_Kur_ at slow and fast drug-binding kinetics (Figures 2B,G). Significant APD prolongation is only seen for intermediate drug-binding kinetics (0.3–30 s^−1^ for the open state blocker and 1–30 s^−1^ for the open and inactivated state blocker), and goes well beyond the little APD prolongation resulting from a constant 50% reduction in G_Kur_. However, even the maximal APD prolongation produced by an O or O & I inhibitor in cAF is still ~50 ms less than the APD in nSR in drug-free conditions, which we interpret to suggest that such drugs would have limited toxicity at 1-Hz pacing rate.

At 3-Hz pacing, the two types of inhibitors cause stronger relative prolongation as compared to 1-Hz pacing across the same range of drug-binding kinetics (Figures 2D,I). Notably, all simulated drugs caused APD prolongation at 3-Hz pacing, but the maximal prolongation produced by these theoretical inhibitors did not match the APD prolongation caused by a 100% reduction in G_Kur_. However, drugs with intermediate drug-binding kinetics (3–30 s^−1^ for the O blocker and 10–30 s^−1^ for the O & I blocker) did extend the APD at 3-Hz pacing above the APD in nSR conditions given no block of I_Kur_. Thus, even though G_Kur_ is reduced by 50% in cAF as compared to nSR, Figure 2 illustrates that I_Kur_ inhibitors can still prolong APD in cAF, particularly at 3-Hz pacing.

Figures 2C,H,E,J display the closed (red), open (blue), inactivated (green), and drug-bound (gray) state occupancies during the steady-state AP for the slowest (0.01 s^−1^), intermediate (3 s^−1^), and fastest (100 s^−1^) drug-binding rates. In general, for the slowest drug-binding kinetics, the inhibitors do not bind readily during the AP, and the drug-bound state stays level below 0.4. At intermediate drug-binding kinetics, the inhibitors bind readily during the AP, thus significantly shrinking the open state occupancy. In addition, the off-rate of drug binding is slow enough to achieve maintenance in the drug-bound state during the AP. This allows for considerable AP prolongation, almost mimicking complete block of I_Kur_. Finally, for the fastest drug-binding kinetics, the drugs again bind readily during the AP, but the off-rate of drug binding is so fast as to cause cycling between the drug-free open state and the drug-bound open state during a single AP. This results in prolongation of the drug-free open state occupancy later in the AP that limits AP prolongation. These results are consistent with our previous simulations in nSR. However, given the more positive plateau in the cAF cardiomyocyte AP, K_V_1.5 channels stay open longer, and inactivate more markedly (especially at 3-Hz pacing) as compared to nSR (Figure S7).

**Figure 3:**
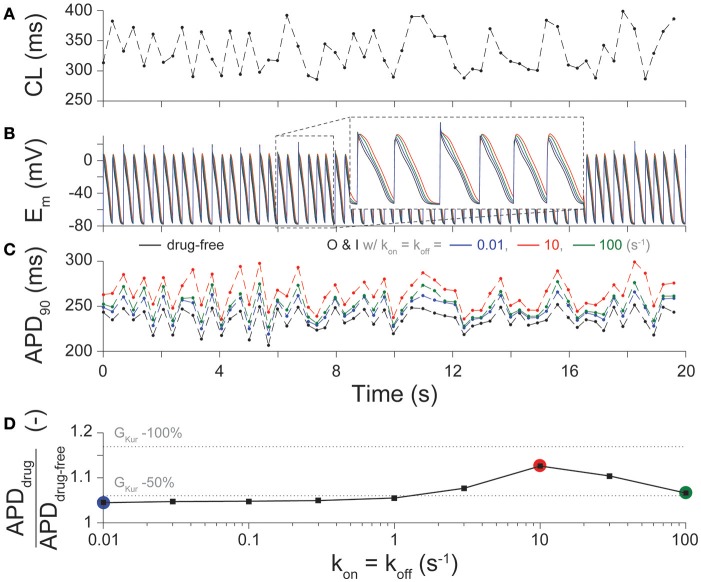
Effect of drug-binding kinetics on APD during irregular pacing**. (A)** Beat-to-beat changes in CL during a 20-s irregular pacing protocol and the resultant time-course of **(B)** E_m_, and **(C)** APD_90_ are shown in cAF cardiomyocytes in drug-free conditions (black), and for O & I blockers with slow (0.01 s^−1^, blue), intermediate (10 s^−1^, red), and fast (100 s^−1^, green) drug-binding kinetics, given k_on_ = k_off_. **(D)** summarizes the percent prolongation (mean APD_90_ after application of drug divided by mean APD_90_ in drug-free conditions during the simulation) for nine different rates of binding kinetics between 0.01 and 100 s^−1^ using half-logarithmic increments, whereby k_on_ = k_off_, K_d_ = 1 μM. These results are compared to 50, and 100% reduction in G_Kur_ (dotted lines) given the same irregular pacing protocol in **(A)**.

Given not only the rapid, but irregular electrical activity seen with AF, we sought to determine how the kinetics of drug binding of I_Kur_ inhibitors affected the time course of E_m_ (Figure 3B) and APD_90_ (Figure 3C) in cAF cardiomyocytes with a randomly variable CL (Figure 3A). Results in drug-free conditions and for an O & I blocker (modeled as in Figure 2F, black) with k_on_ = k_off_ (K_d_ = 1 μM) in Figure 3 again demonstrate a biphasic relationship between drug-binding kinetics and average APD_90_ (Figure 3D), as seen with constant pacing (Figure 2I). Thus, for all future simulations, we used a constant pacing interval that can more easily be standardized in a high-throughput drug-screening process.

### Effect of conformational state specificity and binding/unbinding kinetics on human atrial cardiomyocyte ERP at normal and fast pacing rates in cAF conditions

**Figure 4:**
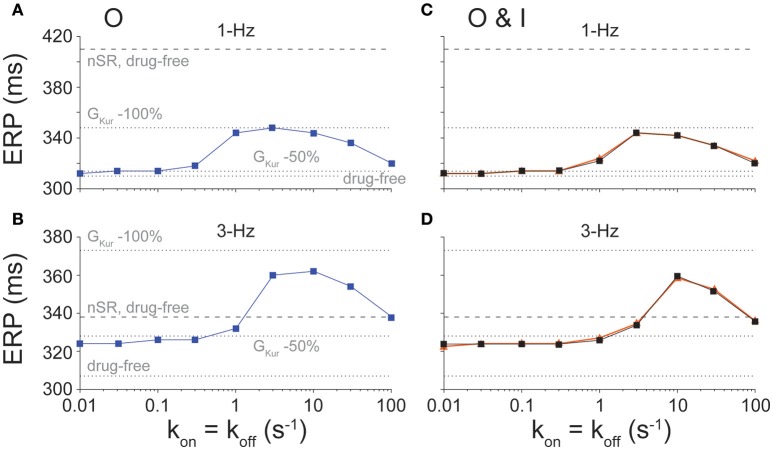
Effect of state-dependence and kinetics of drug binding on ERP. ERP was determined for open (**A**, 1-Hz and **B**, 3-Hz pacing rate) and open and inactivated (**C**, 1-Hz and **D**, 3-Hz pacing rate) state blockers given nine different rates of binding kinetics between 0.01 and 100 s^−1^ using half-logarithmic increments, whereby k_off_ = k_on_, K_d_ = 1 μM. For O & I blockers, we either allowed or prevented transitions between drug-bound states (orange vs. black traces in **C,D**). Simulations were also run in nSR and cAF drug-free conditions, and in cAF given a 50 and 100% reduction in G_Kur_.

The desired effect of I_Kur_ inhibitors is prolongation of atrial ERP (Amos et al., 1996; Christ et al., 2008; Sanchez et al., 2012; Loose et al., 2014; Ford et al., 2016), particularly during fast pacing rates typifying AF. Thus, we assessed the effects of binding/unbinding kinetics on the ERP for O (Figures 4A,B) and O & I (Figures 4C,D) blockers. Simulations reveal a similar biphasic relationship between ERP and drug-binding kinetics at 1- and 3-Hz pacing for both types of inhibitors, which mirror the drugs' effects on APD (Figure 2).

At 1-Hz pacing, I_Kur_ inhibitors cause minimal ERP prolongation at slow drug-binding rates (≤0.3 s^−1^ for O blockers and ≤1 s^−1^ for O & I blockers) and fast drug-binding rates (100 s^−1^). Although substantial ERP changes are predicted at intermediate drug-binding rates (1–30 s^−1^ for O blockers and 3–30 s^−1^ for O & I blockers), ERP prolongation remains ~62 ms lower than the ERP in nSR given no block of I_Kur_ for both inhibitors.

At 3-Hz pacing, however, I_Kur_ inhibitors appear to be more effective at extending ERP than APD, which is a favorable drug property as previously demonstrated for Class I antiarrhythmic drugs which cause clinically relevant post-repolarization refractoriness. For all drug-binding kinetics, ERP prolongation is at least equivalent to that caused by a constant 50% reduction in G_Kur_ (Figures 4B,D). Notably, for intermediate drug-binding kinetics (3–30 s^−1^ for O inhibitors and 10–30 s^−1^ for O & I inhibitors), drug-induced ERP prolongation extends above the ERP in nSR in drug-free conditions, and the fastest drug-binding kinetics prolong the ERP to a point that closely resembles that in nSR in drug-free conditions. These drugs showing substantial ERP prolongation at 3-Hz pacing in cAF (with APD at slow pacing rates being well below that in nSR, see Figure 2) might represent suitable compounds for AF-selective therapy.

### Effects of drug binding/unbinding kinetics with variable K_d_ on APD, ERP, and Ca^2+^ handling

**Figure 5:**
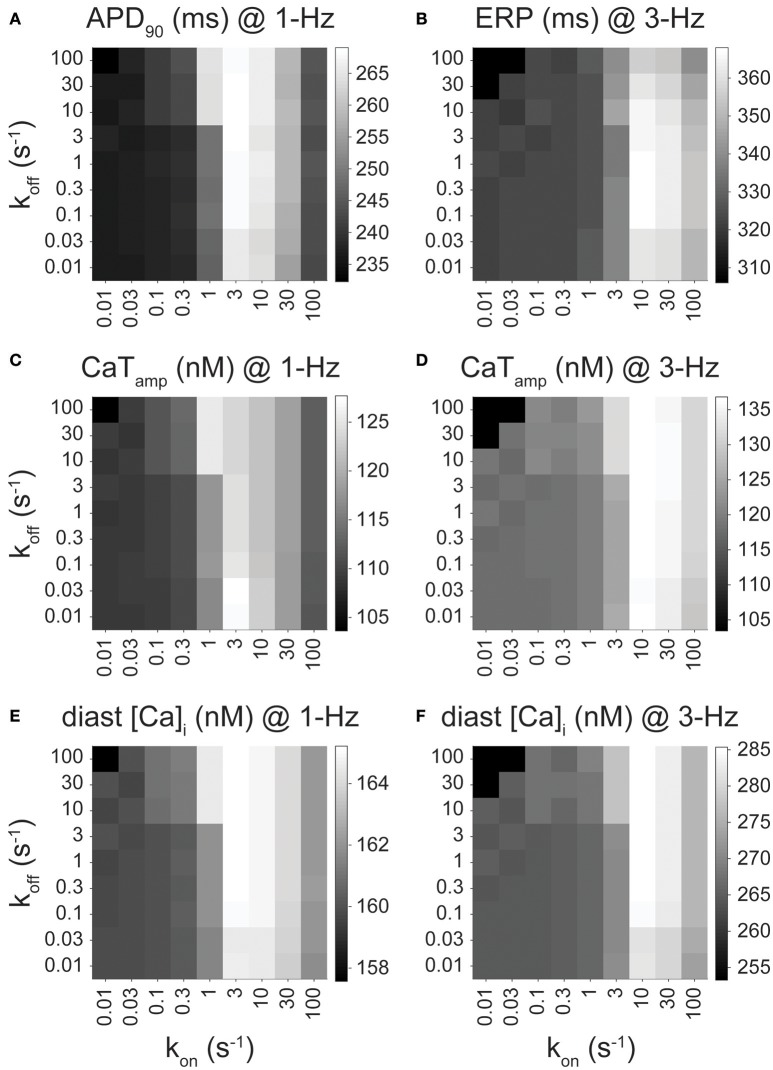
Effect of drug-binding kinetics on APD_90_, ERP, CaT_amp_, and diastolic [Ca^2+^]_i_ for an open and inactivated state blocker. **(A)** APD_90_ (at 1 Hz), **(B)** ERP (at 3 Hz), **(C,D)** CaT_amp_ (at 1 and 3 Hz), and **(E,F)** diastolic [Ca^2+^]_i_ (at 1 and 3 Hz) are plotted for open and inactivated state blockers with varying binding kinetics, which were simulated via permutations of nine different drug-binding rates of (from 0.01 to 100 s^−1^) while keeping k_on,O_ = k_on,I_ and k_off,O_ = k_off,I_. CaT_amp_ is 103.6, 109.4, and 120.4 nM at 1 Hz, and 103.4, 120.4, and 135.9 nM at 3 Hz for drug-free, 50 and 100% I_Kur_ block, respectively. Diastolic [Ca^2+^]_i_ is 157.6, 160.0, and 165.2 nM at 1 Hz, and 253.3, 266.9, and 286.9 nM at 3 Hz for drug-free, 50 and 100% I_Kur_ block, respectively.

Figures 2, 3, 4 show the results from drug scenarios where the on- and off-rate of drug binding are equal to one another (k_on_ = k_off_, K_d_ = 1 μM), but even closely related I_Kur_ inhibitors can have dissimilar K_d_ values (Lagrutta et al., 2006). Thus, we simulated all permutations of the nine different rates of drug binding (0.01 to 100 s^−1^), yielding 81 different combinations of k_on_ and k_off_ for the O & I state inhibitors (assuming equal affinities for open and inactivated states) at 1- and 3-Hz pacing. We assessed the effects of these drugs (at their IC_50_ concentration) on APD, ERP, CaT_amp_, and diastolic [Ca^2+^]_i_. Figure 5 shows the output of the simulations for an O & I inhibitor (modeled as in Figure 2F, black) in the form of a heatmap, where the diagonals of the squares from the bottom left to the top right corner correspond to drug scenarios where k_on_ = k_off_ (K_d_ = 1 μM). Except for the drugs with the largest K_d_ values (k_off_ >> k_on_), when k_on_ is held constant, APD, ERP, and Ca^2+^ handling are not very sensitive to changes in k_off._ Thus, the effects of I_Kur_ inhibitors on atrial electrophysiology and Ca^2+^ handling are largely driven by k_on_ rates as compared to k_off_ rates.

In cAF conditions, ideal I_Kur_ inhibitors exhibiting AF-selectivity will prolong atrial refractoriness (ERP prolongation at 3-Hz pacing), have limited toxicity (minimal to no APD prolongation at 1-Hz pacing), and have a positive inotropic effect (an increase in CaT_amp_ at 1-Hz pacing). O & I inhibitors with a large K_d_ do not display any of the desired favorable drug properties including prolongation of ERP at 3-Hz pacing (Figure 5B) or increase in CaT_amp_ (Figures 5C,D), as their effects on APD, ERP, and Ca^2+^ handling are minimal, resembling drug-free conditions. Intermediate k_on_ rates (3–30 s^−1^ for 1-Hz pacing and 10–30 s^−1^ for 3-Hz pacing) cause the most significant increase in all the outputs displayed in Figure 5. For example, drugs with a k_on_ rate equal to 10 s^−1^ cause the greatest ERP prolongation at 3-Hz pacing (Figure 5B) and increase in CaT_amp_ and diastolic [Ca^2+^]_i_ (Figures 5C,E). Note, there is also significant APD prolongation at 1-Hz pacing when k_on_ is in the intermediate drug-binding range (Figure 5A), but none of the 81 permutations of the simulated open and inactivated state inhibitor cause the APD to get close to the APD in nSR at 1-Hz pacing (320 ms). Thus, the APD prolongation seen in Figure 5A does not necessarily disqualify any of these theoretical drug candidates for AF therapy. Likewise, at 3-Hz pacing, the increase in CaT_amp_ and diastolic [Ca^2+^]_i_ mirrors the prolongation in APD and ERP at 3-Hz pacing (Figures 5D,F). While an excessive increase in diastolic [Ca^2+^]_i_ might be deleterious, we find it to remain well below the predicted value in the nSR human atrial cardiomyocyte model (~360 nM).

In our previous study in nSR (Ellinwood et al., 2017), we found that O & I inhibitors with the fastest drug-binding kinetics (30–100 s^−1^) cause ERP prolongation at 3-Hz pacing and no APD prolongation at 1-Hz pacing. These same inhibitors display favorable fast pacing-rate selectivity in atrial cardiomyocytes from cAF according to our simulations shown in Figures 5A,B. However, if we are not as concerned with APD prolongation in cAF conditions at 1-Hz pacing, then drugs with a k_on_ rate in the intermediate drug-binding range (3–30 s^−1^) would also be efficacious and perhaps more efficacious since they cause a positive inotropic effect at 1-Hz pacing (Figures 5C,E).

### Effect of relative state-specific drug binding

**Figure 6:**
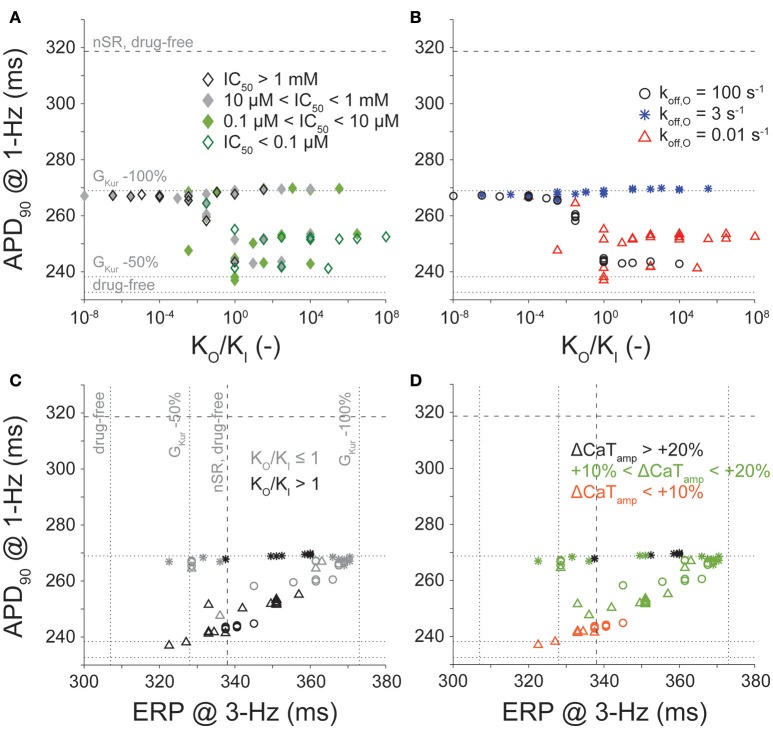
Effect of conformational state affinity and drug-binding kinetics of an open and inactivated state blocker on APD_90_, ERP, and Ca^2+^ handling. Open and inactivated state I_Kur_ blockers with varying affinities to the open and inactivated states were simulated via permutations of three different rates of binding kinetics (0.01, 3, and 100 s^−1^). Simulations were equilibrated for 300 beats at 1-Hz pacing or 900 beats at 3-Hz pacing using a [drug] equal to the IC_50_ value. **(A,B)** report APD_90_ values (at 1 Hz) plotted as a function of the ratio of the open to the inactivated state affinity (K_O_/K_I_) used in each simulation. **(C,D)** report APD_90_ (at 1 Hz) and ERP values (at 3 Hz). Color code in **(A)** is for IC_50_ levels. Symbols in **(B,C,D)** indicate various k_off,O_. Shades in **(C)** reflect either higher affinity to the open or the inactivated state. Color code in **(D)** corresponds to the variable degree of CaT_amp_ increase (at 1 Hz) induced by I_Kur_ block. Horizontal and vertical lines represent APD_90_ and ERP values obtained in cAF in drug-free conditions, and 50 and 100% reduction in G_Kur_ (dotted lines), and in nSR in drug-free conditions (dashed lines).

Because many I_Kur_ inhibitors bind to multiple states of K_V_1.5 with variable affinity (Bouchard and Fedida, 1995; Lagrutta et al., 2006; Ford et al., 2016), we allowed k_on_ for the open state (k_on,O_), k_off_ for the open state (k_off,O_), k_on_ for the inactivated state (k_on,I_), and k_off_ for the inactivated state (k_off,I_) to have any of the three binding rates (0.01, 3, and 100 s^−1^), and varied them independently to yield 81 different drug combinations. We studied the effects of these I_Kur_ blockers in cAF conditions using a [drug] equal to their IC_50_ value at 1-Hz pacing for APD and 3-Hz pacing for ERP. Then, we compared the outputs of APD and ERP to no block, 50, and 100% reduction in G_Kur_ in cAF conditions (Figure 6, dotted lines), along with no block in nSR conditions (Figure 6, dashed lines).

Figures 6A,B display the relationship between APD (at 1-Hz pacing) and K_O_/K_I_. Data points in Figure 6A are separated by IC_50_ cutoffs of 0.1 μM, 10 μM, and 1 mM, and show that when K_O_/K_I_ < 1, we almost always obtain maximal AP prolongation (this also corresponds to larger IC_50_ values). In Figure 6B, we separated the points according to the drug's k_off,O_ rate (0.01, 3, or 100 s^−1^), which revealed that when K_O_/K_I_ > 1, we only obtain significant AP prolongation when k_off,O_ is equal to 3 s^−1^ (i.e., the intermediate drug-binding rate). These results in the cAF-remodeled atrial cardiomyocyte correspond well with the results from our previous study of I_Kur_ inhibitors in nSR (Ellinwood et al., 2017). Nevertheless, none of the 81 simulated O & I inhibitors in Figure 6 prolong the AP beyond the APD found in nSR at 1-Hz pacing.

Figures 6C,D present the relationship between APD at 1-Hz pacing and ERP at 3-Hz pacing for the O & I inhibitors with a variable K_O_/K_I_ ratio. In Figure 6C, light gray symbols correspond to K_O_/K_I_ ≤ 1, and dark symbols correspond to K_O_/K_I_ > 1). The O & I blockers displaying favorable pacing-rate selectivity, i.e., producing ERP prolongation at 3-Hz pacing while having moderate effect on APD (and ERP) at 1-Hz pacing, are the ones with K_O_/K_I_ > 1, except if k_off,O_ equals 3 s^−1^. However, as none of the 81 simulated O & I inhibitors in Figure 6 prolong the APD beyond that found in nSR at 1-Hz pacing, one could argue that none of the drugs is expected to cause harmful AP prolongation when AF is terminated. To try and enrich our metric, in Figure 6D we also categorize the drugs according to percent increase in CaT_amp_. The best-performing drugs will cause ERP prolongation at 3-Hz pacing in cAF (above nSR), and have a positive inotropic effect (Figure 6D, black). Corresponding with the results showcased in Figure 5, drugs with intermediate binding rates (e.g., k_off,O_ = 3 s^−1^) may thus be favorable given their stronger inotropic effect.

## Discussion

**Figure 7:**
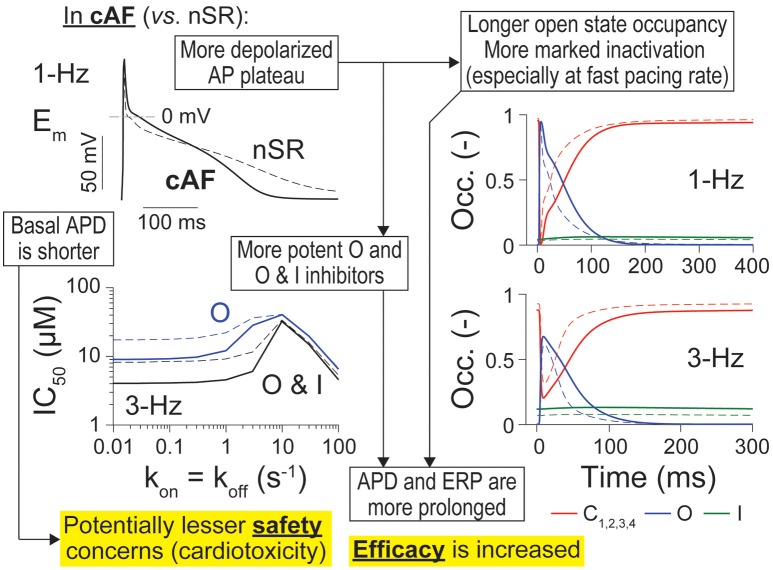
Summary of main findings. Atrial cardiomyocytes in cAF (solid lines) vs. nSR (dotted lines) have different AP trajectories, including a shorter APD and more depolarized plateau (top left). The latter causes longer open state occupancy (right, blue solid vs. dashed lines) and stronger inactivation of the channel in cAF conditions (especially at fast pacing rates, right, green solid vs. dashed lines). I_Kur_ inhibitors appear also more potent in cAF vs. nSR (bottom left). These factors render APD and ERP more sensitive to inhibition by O and O & I inhibitors of K_V_1.5, thus increasing efficacy of these drugs in cAF vs. nSR. Because basal APD is shorter in cAF, there are potentially less safety concerns due to drug-induced AP prolongation and subsequent afterdepolarization-driven proarrhythmia.

In this study, we sought to determine if I_Kur_ is a suitable anti-AF target despite it being downregulated in cAF patients, and, if so, what are the kinetic and state-dependent binding properties that maximize anti-AF efficacy and limit potential cardiotoxicity. Building off our previous study in nSR conditions (Ellinwood et al., 2017), we implemented an *in silico* assessment of I_Kur_ inhibitors in cAF atrial cardiomyocyte models, and identified metrics for delineating ideal K_V_1.5 blockers against AF. Our results point to I_Kur_ inhibition as a valid strategy to prolong atrial refractoriness also generating a positive inotropic effect in cAF conditions. Although increasing force generation may not be a useful therapeutic goal at the high atrial rates seen during AF, it can be important to counteract atrial hypocontractility after cardioversion of AF to nSR. Interestingly, our simulations suggest that electrophysiological properties in cAF cardiomyocytes, such as shorter AP and more depolarized plateau potential, both might act to increase efficacy and dampen cardiotoxicity of potential K_V_1.5-targeting drugs as compared to nSR (Ellinwood et al., 2017; Figure 7).

### I_Kur_ role in APD and ERP regulation is preserved despite its downregulation in cAF

Figure S7 shows the differences in the time courses of E_m_, I_Kur_, and closed, open, and inactivated state occupancies of K_V_1.5 in cAF and nSR during the AP. Despite the reduced peak current, the channel stays open later in cAF (at both 1- and 3-Hz pacing) because of the more depolarized AP plateau. Thus, the consequences of I_Kur_ inhibition, including the extent of AP and ERP prolongation, depend not only on I_Kur_ magnitude (i.e., maximal conductance), but also on other fluxes affected by AF-induced remodeling, which affect E_m_ and thus E_m_-dependent properties of I_Kur_ (Figure 7). For example, our group and others have hypothesized that the extent of AP and ERP prolongation due to I_Kur_ blockade depends on the AF-induced remodeling of other K^+^ currents (Lagrutta et al., 2006; Morotti et al., 2016a; Aguilar et al., 2017; Colman et al., 2017), and relative strengths of I_CaL_ and I_Kur_ (Wettwer et al., 2004; Grandi and Maleckar, 2016). Our sensitivity analysis (Figure 1C and Figures S1–S6) revealed that APD_90_ and ERP are more sensitive to changes in G_Kur_ at fast vs. slow pacing rates. Aguilar et al. recently determined that the relative contribution of I_Kur_ to AP repolarization increases at higher frequencies because of reduced activation of the rapid delayed-rectifier current I_Kr_ (Aguilar et al., 2017). Our results concur with these findings, as our sensitivity analysis shows that APD_90_ and ERP are less sensitive to changes in G_Kr_ at 3-Hz pacing as compared to 1-Hz pacing in nSR conditions (Figures S2, S3). Most importantly, we also found that G_Kur_ impacted the duration of AP repolarization and refractoriness more in cAF vs. nSR (even though this parameter was halved in the cAF model) at 3-Hz, but not at 1-Hz pacing (i.e., fast pacing-rate selectivity). This is a favorable drug property to avoid harmful AP prolongation (which is also limited by the reduced basal APD) if AF is terminated. Similar to Aguilar et al. (2017), our results suggest that the APD- (and ERP)-prolonging effect of I_Kur_ block is not affected by I_Kur_ downregulation.

### Enhanced efficacy and safety of I_Kur_ inhibitors in cAF vs. nSR

We focused here on O and O & I blockers because we have previously shown that these inhibitors display fast pacing-rate selectivity in nSR (Ellinwood et al., 2017). This choice was also supported by the increased occupancy of open and inactivated states in cAF conditions (Figure 7). In our previous report in nSR (Ellinwood et al., 2017), we found that when k_on_ = k_off_ (i) slow drug-binding kinetics caused minimal APD changes and modest ERP prolongation; (ii) intermediate drug-binding kinetics led to substantial AP and ERP prolongation; and (iii) fast drug-binding kinetics failed to produce substantial AP or ERP prolongation at normal pacing rate, but increased the ERP at 3-Hz pacing. While in cAF the overall biphasic relationship between APD/ERP and drug-binding kinetics was maintained (see Figures 2–4), notably, at 1-Hz pacing rate, even the maximal AP prolongation induced by I_Kur_ inhibition in cAF is not sufficient to reach the APD observed in nSR in drug-free conditions. This might indicate that there are less safety concerns for K_V_1.5 block in cAF patients. At 3-Hz pacing, ERP prolongation is at least equivalent to that caused by a constant 50% reduction in G_Kur_, and, for intermediate and fast drug-binding kinetics, the ERP is equal to or greater than the one obtained in nSR in drug-free conditions. These observations suggest that O and O & I inhibitors have a broader range of efficacy in cAF vs. nSR. We assessed whether closed state inhibitors, which displayed reverse-rate dependence in terms of potency (Ellinwood et al., 2017), may also be effective and safe anti-AF agents in cAF conditions (Figure S8). We found that these blockers prolong ERP at 3-Hz pacing (Figure S8G) while minimally prolonging the cAF AP at 1-Hz pacing at the fastest drug-binding kinetics (≥30 s^−1^, Figure S8B). However, they had a smaller maximal effect and kinetic range for prolonging the ERP at 3-Hz pacing beyond nSR conditions as compared to O and O & I blockers.

We enriched our metric for quantifying anti-AF efficacy and safety of I_Kur_ inhibitors by also accounting for changes in Ca^2+^-handling parameters, namely CaT_amp_ and diastolic [Ca^2+^]_i_ (Tsujimae et al., 2008; Cavero and Holzgrefe, 2014; Lancaster and Sobie, 2016; Li et al., 2017), which provided additional detail to refine the search for best-performing drugs. In identifying the ideal drug characteristics, we looked for inhibitors that prolong ERP (especially at fast pacing rates), limit APD prolongation at slow pacing-rates, and improve atrial inotropy, i.e., increase CaT_amp_. Increasing force generation might be a useful outcome after cardioversion to nSR.

When K_O_ = K_I_, the best-performing O & I inhibitors were those with intermediate k_on_ rates (3–30 s^−1^), because they prolonged ERP at 3-Hz pacing and increased CaT_amp_ and diastolic [Ca^2+^]_i_ at 1-Hz pacing (Figure 5). These inhibitors also prolonged the AP at 1-Hz pacing and increased CaT_amp_ and diastolic [Ca^2+^]_i_ at 3-Hz pacing—thus potentially predisposing to harmful AP prolongation and Ca^2+^ overload. However, we note that such cardiotoxicity is unlikely considering the fact that the maximum increases of APD and CaT_amp_ still remain far below the corresponding values obtained in nSR in drug-free conditions. In our previous study, we highlighted that the best-performing drugs in nSR were the O & I inhibitors with the fastest drug-binding kinetics (Ellinwood et al., 2017). While these drugs are still efficacious at prolonging ERP at 3-Hz pacing in cAF, they have limited effect on Ca^2+^ handling.

When K_I_ and K_O_ were varied, the relationships between APD at 1-Hz pacing and affinity ratio (K_O_/K_I_) are similar to those in nSR (Figures 6A,B; Ellinwood et al., 2017), except none of the 81 simulated O & I inhibitors prolonged the AP beyond the duration found in nSR in drug-free condition. Likewise, the relationship between APD at 1-Hz pacing and ERP at 3-Hz pacing is similar to nSR (Figures 6C,D; Ellinwood et al., 2017), but none of the drugs exhibit obvious toxicity. The same O & I inhibitors simulated in cAF conditions were more effective at prolonging ERP at 3-Hz pacing rates as compared to nSR conditions. Thus, on average, the same inhibitors in Figure 6 exhibit less toxicity and greater efficacy in cAF vs. nSR.

In their simulation study, Aguilar et al. concluded that the ability of (simple pore) I_Kur_ block to terminate simulated AF was greatly attenuated by remodeling, because the block-induced AP prolongation was insufficient to counteract the strong effects of cAF-induced remodeling (Aguilar et al., 2017). Notably, here we show that depending on the drug-binding kinetics, certain I_Kur_ inhibitors can markedly counteract the effect of cAF-associated remodeling, and bring AP and ERP parameters close to nSR values, i.e., have a greater effect than simple pore blockers.

### Limitations and future directions

We presented a theoretical study of the effects of I_Kur_ inhibitors in cAF, and compared our results to our previous study in nSR atrial cardiomyocytes. We acknowledge several limitations to the described approach, which provide opportunities for further extensions. First, we only considered direct drug effects on K_V_1.5, and future analysis should consider multi-channel effects of I_Kur_ inhibitors (Ford and Milnes, 2008; Li et al., 2017), as this realistically occurs *in vivo* in the clinical setting. We only considered cardiotoxicity at the atrial level, assuming that the absence of I_Kur_ in ventricles prevents ventricular proarrhythmia. However, this might not be true for real I_Kur_ blockers with off-target effects. Here, we simulated I_Kur_ block at the cellular level with no contribution of structural tissue remodeling and defined I_Kur_ inhibitors' efficacy and toxicity by tracking only electrophysiological properties such as APD, ERP, CaT_amp_, and diastolic [Ca^2+^]_i_. While this is an important first step in defining metrics for AF-selectivity, other arrhythmia indices and integration of such simulations into tissue and organ level models would improve our ability to discern best-performing drug characteristics of I_Kur_ inhibitors against AF. Since many antiarrhythmic drugs lose anti-AF efficacy with the progression of the arrhythmia, particularly in patients with atrial cardiomyopathy and comorbidities (Goette et al., 2016), I_Kur_ block might be less efficient against AF in the structurally remodeled atrium. Further studies including 2- and 3-dimensional tissue simulations are needed to address this clinically relevant issue. In addition, machine-learning methods have begun to be implemented to analyze AP metrics after the application of a drug and classify the risk (e.g., torsadogenic risk) of the candidate drug (Lancaster and Sobie, 2016). Such methods can also highlight which ion channels contribute most to such risk. Furthermore, this study revealed that the efficacy and toxicity of I_Kur_ inhibitors is modulated by the extent of atrial ionic remodeling, and likely by the relative expressions of many ion channels and transporters (Figures S1–S6). Thus, given the differences in AP properties and ion channel expression in patients with AF (Heijman et al., 2014), and differences in I_Kur_ remodeling in the right vs. the left atria (Dobrev and Ravens, 2003; Caballero et al., 2010), we hypothesize that certain subpopulations of nSR and cAF patients may be more responsive to therapy with I_Kur_ inhibitors, i.e., degree and heterogeneity of I_Kur_ remodeling in atrial tissue might impact safety and anti-AF efficacy of drugs. Future studies could identify which cell characteristics lead to more favorable responses to anti-I_Kur_ therapy utilizing sensitivity analysis and variations of nSR and cAF models similar to the methods discussed in Figure 1 and in (Sobie, 2009; Lee et al., 2013; Cummins et al., 2014; Devenyi and Sobie, 2015; Morotti and Grandi, 2017). This information could be useful for a personalized (precision) medicine approach to AF treatment or helpful in suggesting potential combination therapies with I_Kur_ inhibitors.

Finally, advancements in high-throughput screening methods (Obergrussberger et al., 2016; Picones et al., 2016; Molokanova et al., 2017) provide functional drug screening capabilities that can be coupled with *in silico* investigations such as the one described here to help identify actual candidate compounds for *in vivo* testing. Such technologies can potentially be implemented to simultaneously screen many K_V_1.5-selective compounds for the desired kinetics, state-dependence, and rate-dependence of I_Kur_ block. In addition, multi-parallel recordings from atrial-like cardiomyocytes from induced human pluripotent stem cells is also emerging as a preclinical model for evaluating drugs targeting atrial-specific ion channels, such as K_V_1.5 (Devalla et al., 2015), particularly in combination with AP-clamp experiments. These could be coupled with *in silico* studies such as this one for delineating the ideal properties of AF-selective drugs and gaining a more comprehensive understanding of the arrhythmic risk of candidate compounds.

## Conclusions

In this study, efficacy and cardiotoxicity on cAF atrial cardiomyocytes of theoretical I_Kur_ inhibitors were assessed *in silico*. We concluded that I_Kur_ is a promising anti-AF target, even if strongly downregulated in cAF condition. We confirmed that steady-state IC_50_ values are insufficient to predict how candidate compounds will interact with a dynamically changing electrophysiological substrate, thus emphasizing the importance of accounting for kinetic and state-dependent drug-binding properties. This approach could aid experimental and screening efforts to identify the complex net impact of I_Kur_ inhibition in different AF-remodeling conditions during the pre-clinical drug development process.

## Author contributions

Designed simulation experiments: NE, SM, EG. Performed modeling and simulations: NE, SM. Wrote the manuscript: NE, DD, SM, EG.

### Conflict of interest statement

The authors declare that the research was conducted in the absence of any commercial or financial relationships that could be construed as a potential conflict of interest.

## Abbreviations

AP: action potential
APD: AP duration
APD_40_: APD to 40% repolarization
APD_90_: APD to 90% repolarization
AF: atrial fibrillation
C: closed state
cAF: chronic AF
CaT: Ca^2+^ transient
CaT_amp_: CaT amplitude
CL: cycle length
EAD: early afterdepolarization
E_m_: membrane potential
ERP: effective refractory period
G_Kur_: maximal conductance of the ultra-rapid delayed-rectifier K^+^ current
I: inactivated state
I_Kur_: ultra-rapid delayed-rectifier K^+^ current
nSR: normal sinus rhythm
O: open state.
