# Supplementary material for: In Silico Assessment of Efficacy and Safety of IKur Inhibitors in Chronic Atrial Fibrillation: Role of Kinetics and State-Dependence of Drug Binding
Source: Front Pharmacol. 2017 Nov 7;8:799. doi: 10.3389/fphar.2017.00799 (PMC5681918; doi:10.3389/fphar.2017.00799)
Supplement: Supplementary file 1 [file Presentation1.PDF]

Supplementary Material

In silico assessment of efficacy and safety of I_{Kur} inhibitors in chronic atrial fibrillation: role of kinetics and state-dependence of drug binding

Nicholas Ellinwood[1], Dobromir Dobrev[2], Stefano Morotti[1], Eleonora Grandi[1]

[1]*Department of Pharmacology, University of California Davis, Davis, CA, USA*
[2]*Institute of Pharmacology, West German Heart and Vascular Center, University Duisburg-Essen, Essen, Germany*

Corresponding author:
Stefano Morotti
Department of Pharmacology
University of California Davis
451 Health Sciences Drive
Tupper Hall rm 2427
Davis, CA 95616, USA
Email: smorotti@gmail.com
Phone: (530) 752-4780
Fax: (530) 752-7710

Parameters varied to generate nSR and cAF model populations

G_{Na}	maximal conductance of the voltage-gated Na^+ current
G_{NaB}	maximal conductance of the background Na^+ current
v_{NKA}	maximal rate of transport of the Na^+/K^+ ATPase
G_{tof}	maximal conductance of the transient outward K^+ current
G_{Kr}	maximal conductance of the rapidly activating delayed rectifier K^+ current
G_{Ks}	maximal conductance of the slowly activating delayed rectifier K^+ current
G_{Kur}	maximal conductance of the ultra-rapid delayed-rectifier K^+ current
G_{Kp}	maximal conductance of the background K^+ current
G_{K1}	maximal conductance of the inward rectifier K^+ current
G_{ClCa}	maximal conductance of the Ca^{2+}-activated Cl^- current
G_{ClB}	maximal conductance of the background Cl^- current
G_{Ca}	maximal conductance of the L-type Ca^{2+} current
G_{CaB}	maximal conductance of the background Ca^{2+} current
v_{PMCA}	maximal rate of transport of the plasmalemmal Ca^{2+} pump
v_{NCX}	maximal rate of transport of the Na^+/Ca^{2+} exchanger
v_{SERCA}	maximal rate of transport of the sarcoplasmic reticulum Ca^{2+} ATPase
v_{RyR}	maximal rate of ryanodine receptors Ca^{2+} release
v_{leak}	maximal rate of Ca^{2+} leak from the sarcoplasmic reticulum

Supplementary Figures

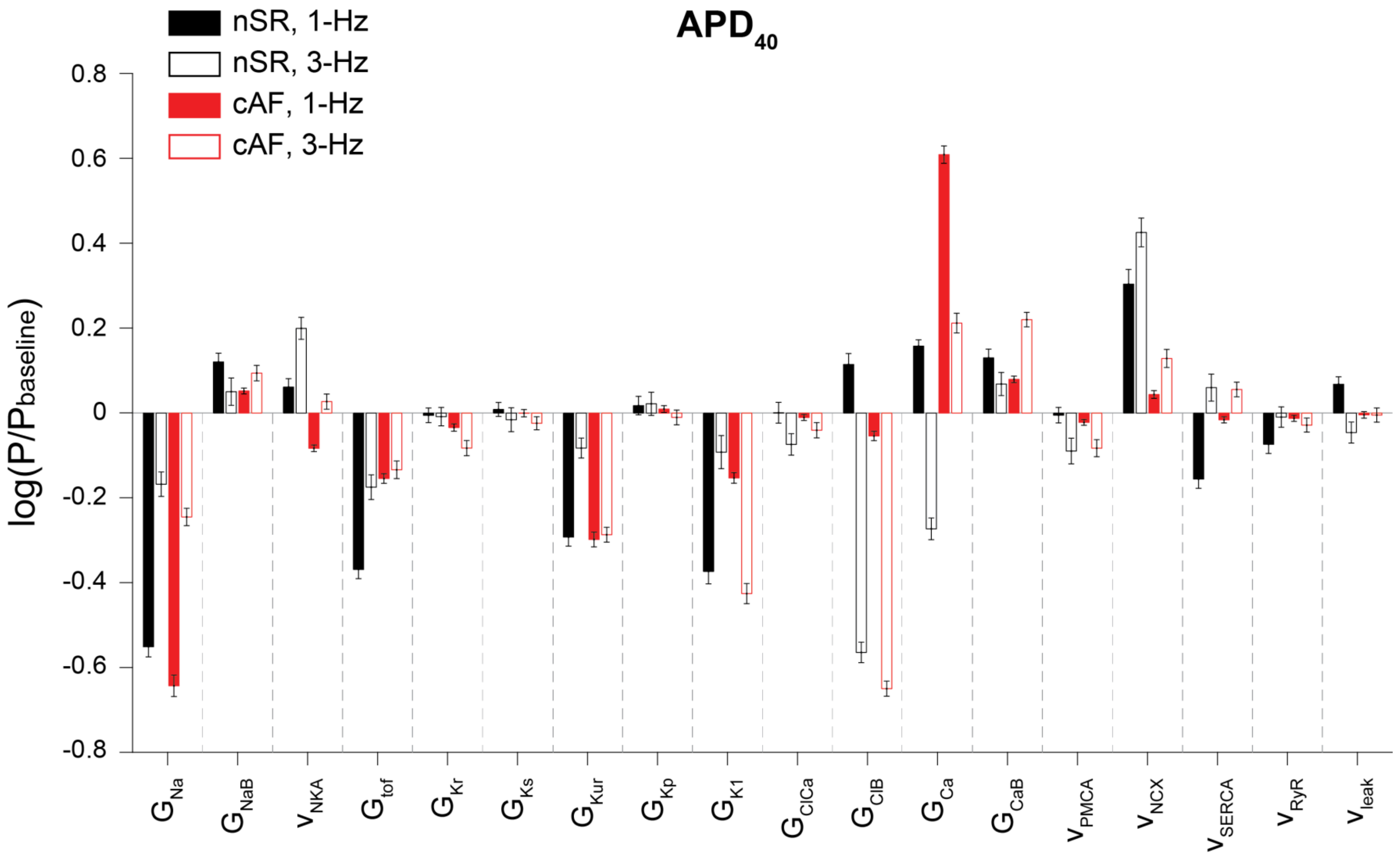

Figure S1. Results of multivariable regression analysis showing how perturbations in model parameters affect APD_{40} during 1- and 3-Hz pacing in nSR and cAF conditions.

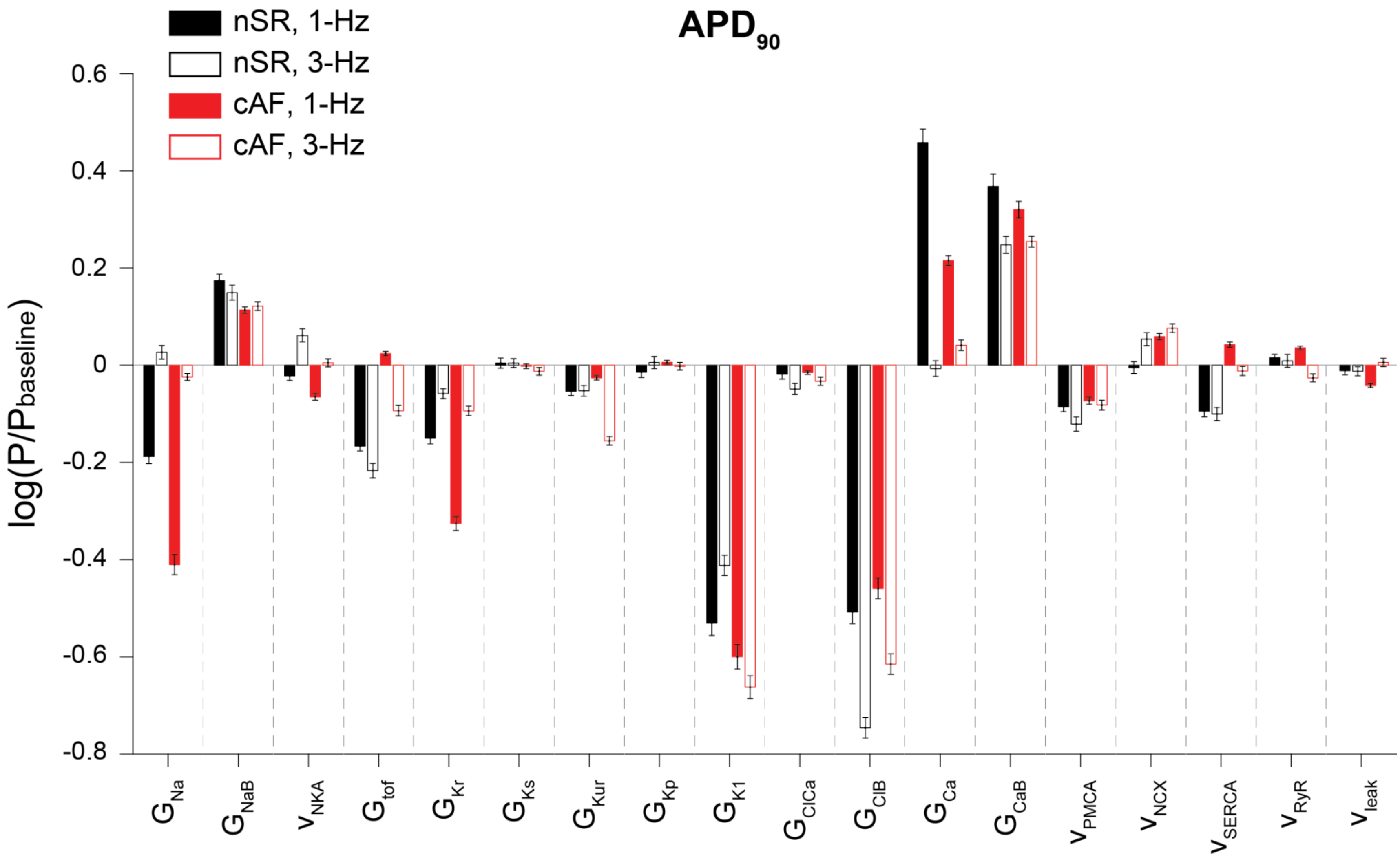

Figure S2. Results of multivariable regression analysis showing how perturbations in model parameters affect APD_{90} during 1- and 3-Hz pacing in nSR and cAF conditions.

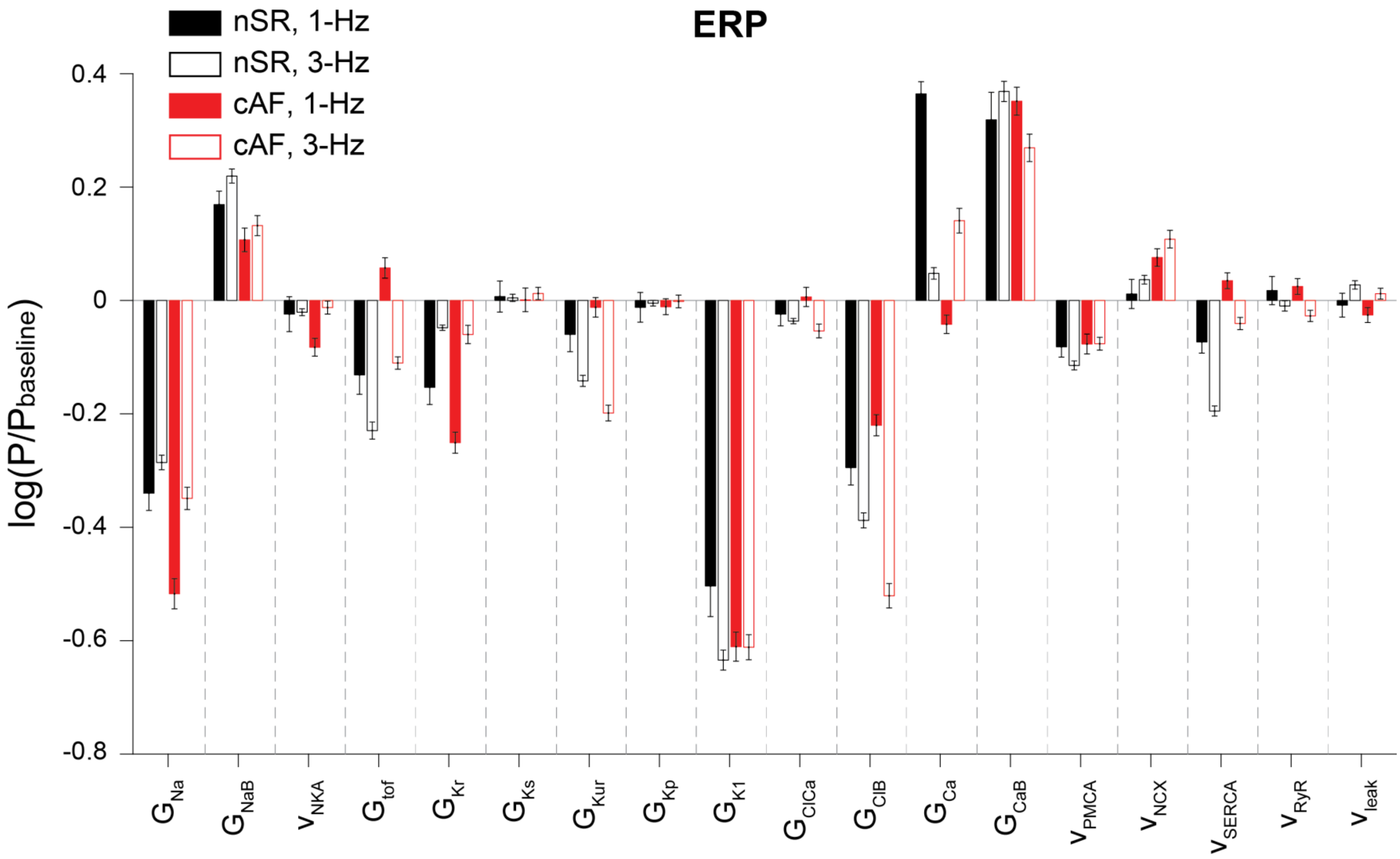

Figure S3. Results of multivariable regression analysis showing how perturbations in model parameters affect ERP during 1- and 3-Hz pacing in nSR and cAF conditions.

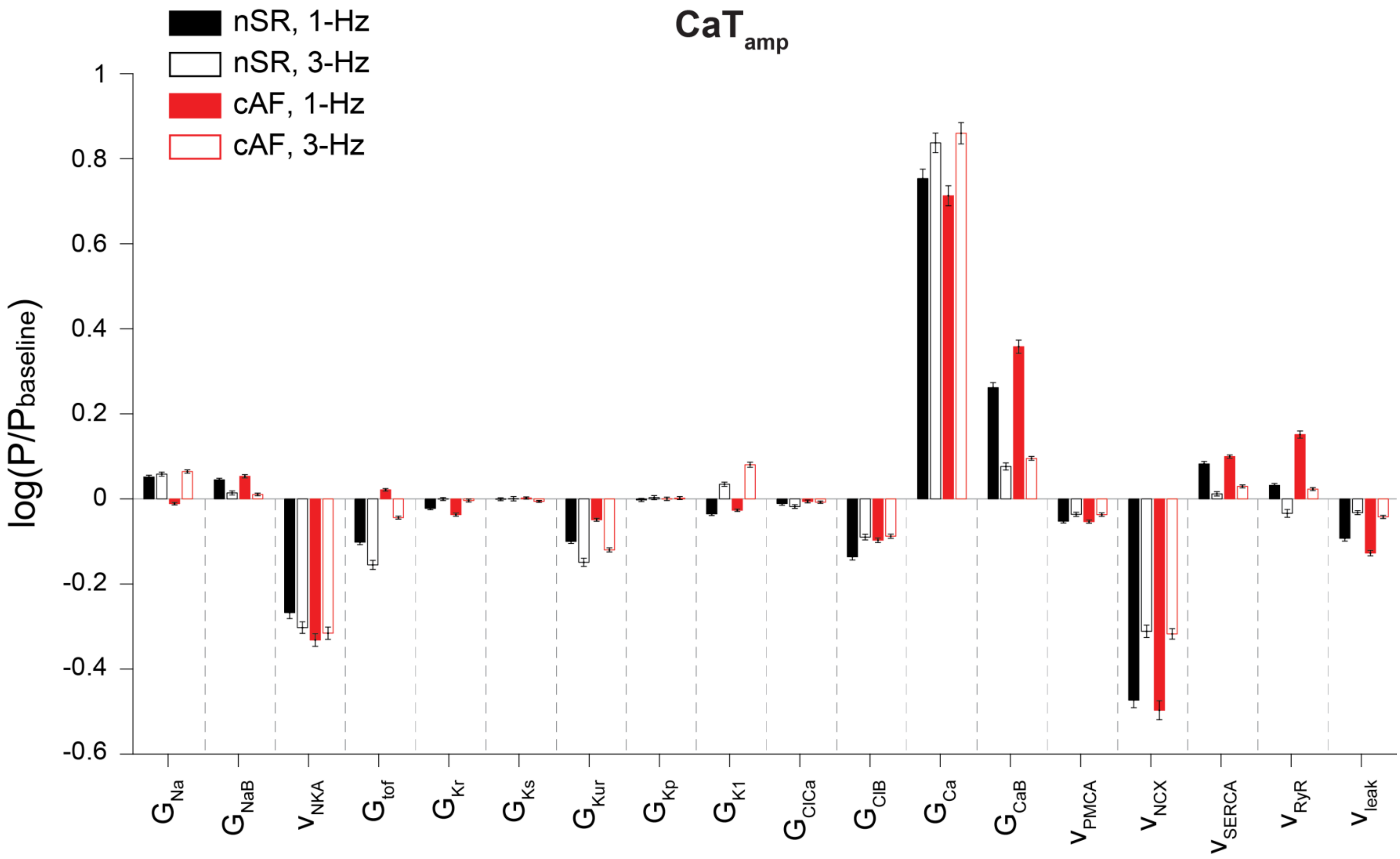

Figure S4. Results of multivariable regression analysis showing how perturbations in model parameters affect CaT_{amp} during 1- and 3-Hz pacing in nSR and cAF conditions.

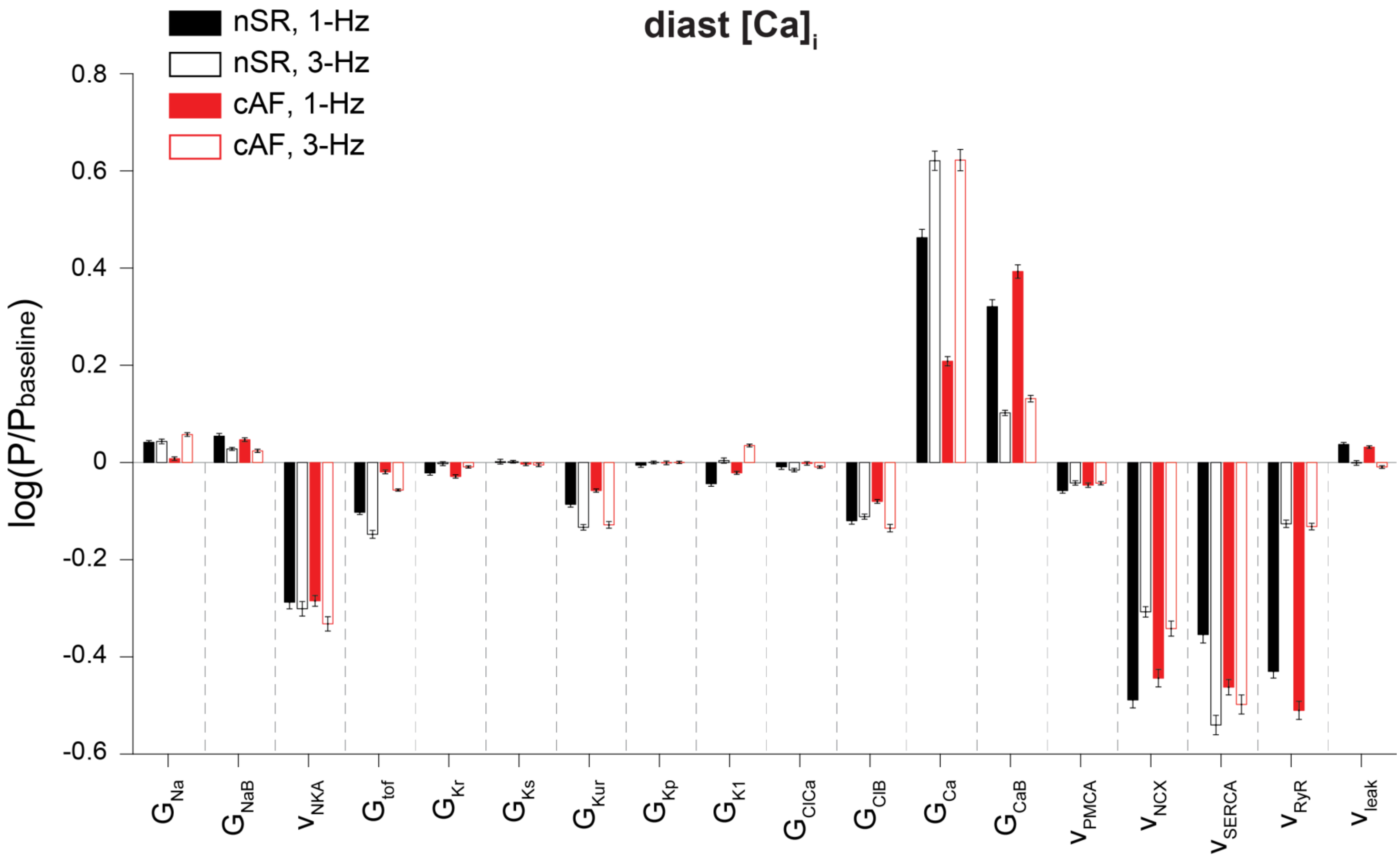

Figure S5. Results of multivariable regression analysis showing how perturbations in model parameters affect diastolic $[Ca^{2+}]_i$ during 1- and 3-Hz pacing in nSR and cAF conditions.

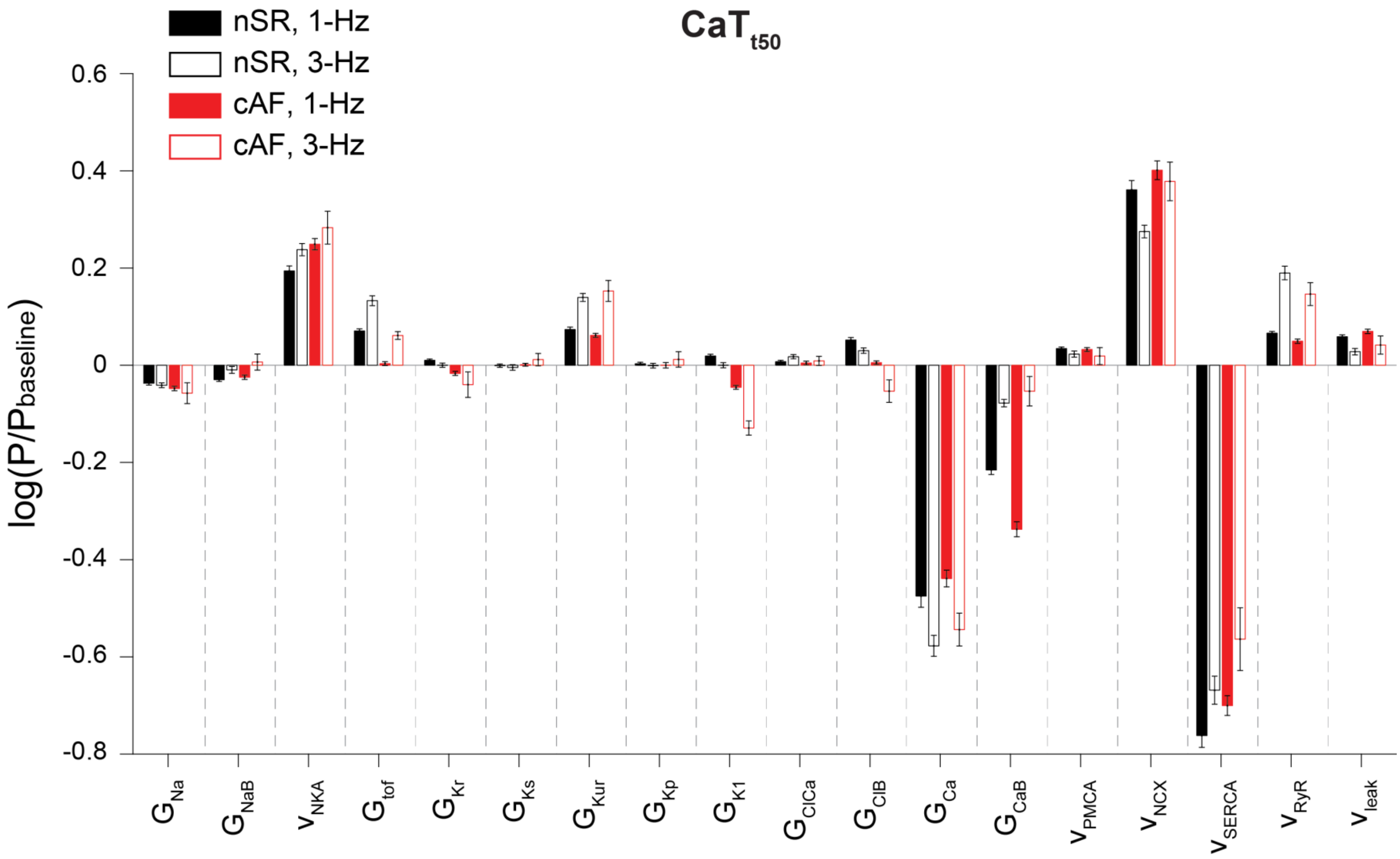

Figure S6. Results of multivariable regression analysis showing how perturbations in model parameters affect the time to 50% CaT decay during 1- and 3-Hz pacing in nSR and cAF conditions.

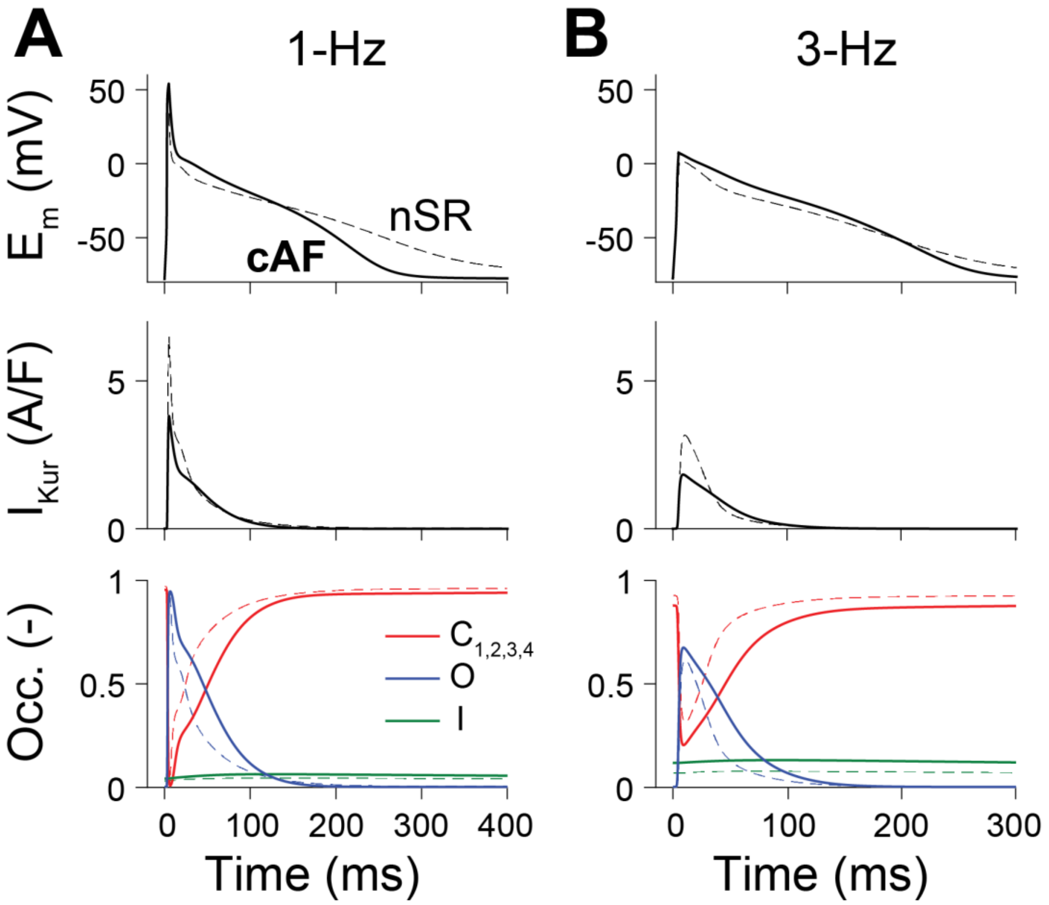

Figure S7. Time courses of E_m (top), I_{Kur} (middle), and closed, open, inactivated state occupancy (bottom) during atrial APs elicited at 1- (**A**) and 3-Hz pacing (**B**). Solid and dashed traces are obtained with the cAF and the nSR models, respectively.

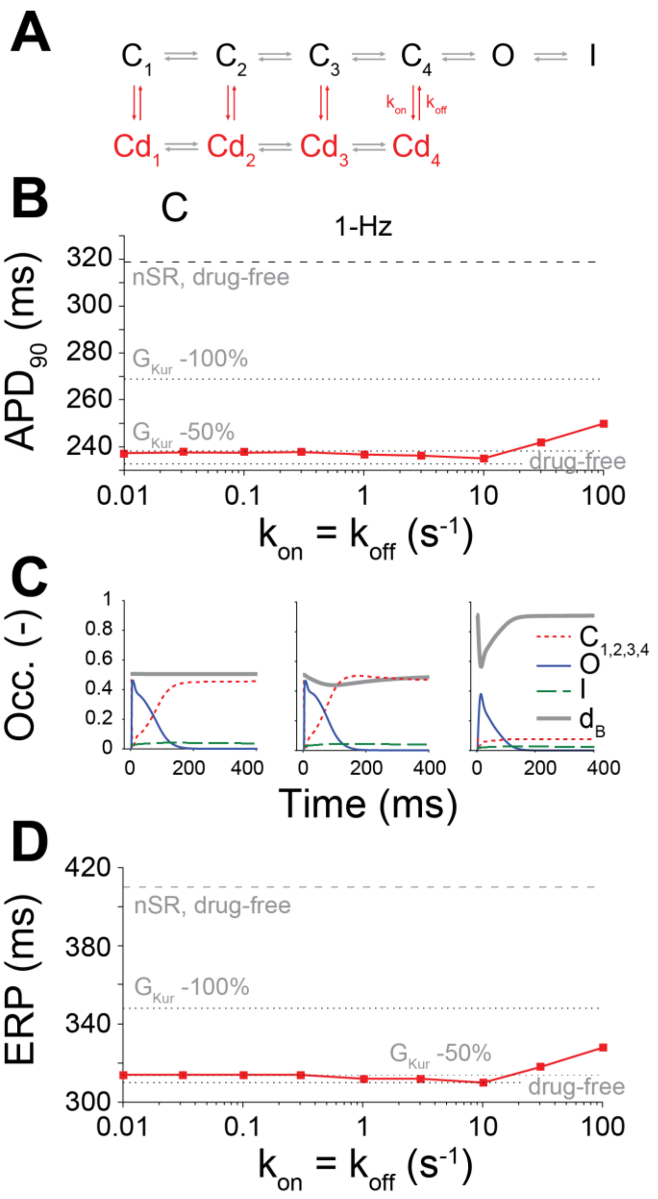

Figure S8. Effect of drug-binding kinetics of closed state blockers on APD and ERP in cAF myocytes. APD_{90} (**B**, 1- and **E**, 3-Hz pacing) and ERP (**D**, 1- and **G**, 3-Hz pacing) were determined for closed state blockers (schematic in **A**) given nine different rates of binding kinetics between 0.01 and 100 s^{-1} using half-logarithmic increments, whereby $k_{off} = k_{on}$, K_d = 1 μM. Simulations were also run in nSR and cAF drug-free conditions, and in cAF given a 50% and 100% reduction in G_{Kur} (dotted and dashed lines in **B**, **E**, **D**, **G**). Simulations were equilibrated for 300 beats at 1-Hz pacing or 900 beats at 3-Hz pacing using a [drug] equal to the IC_{50} value. **C** and **F** show the closed, open, inactivated and drug-bound (d_B, i.e., Cd_1+Cd_2+Cd_3+Cd_4) state occupancies during an AP for three different drug-binding kinetics ($k_{off} = k_{on}$ = 0.01, 3, and 100 s^{-1}).
